# Self-development groups reduce medical school stress: a controlled intervention study

**DOI:** 10.1186/1472-6920-10-23

**Published:** 2010-03-16

**Authors:** Mari Holm, Reidar Tyssen, Kirsten I Stordal, Brit Haver

**Affiliations:** 1 Department of Clinical Medicine, Section for Psychiatry, University of Bergen, N-5020 Bergen, Norway; 2 Department of Behavioural Sciences in Medicine, Institute of Basic Medical Sciences, Faculty of Medicine, University of Oslo, N-0317 Oslo, Norway; 3 Division of Psychiatry, Helse Bergen HF, N-5021 Bergen, Norway

## Abstract

**Background:**

High stress levels and mental health problems are common among medical students and there is a lack of studies on group interventions that aim to reduce such distress during medical school.

**Methods:**

A full class of students (n = 129) participated in group sessions during their third year of medical school in Bergen, Norway. The subsequent third-year class (n = 152) acted as control group, in order to create a quasi-experimental design. Two types of group intervention sessions were offered to the first class. One option was self-development groups led by trained group psychotherapists. Alternatively, students could choose discussion groups that focused on themes of special relevance to doctors, led by experienced general practitioners. The intervention comprised of 12 weekly group sessions each lasting 90 minutes. Data were gathered before the intervention (T1), and three months post intervention (T2). Distress was measured using the Perceived Medical School Stress (PMSS) and Symptom Check List-5 (SCL-5) assessments.

**Results:**

The intervention group showed a significant reduction in PMSS over the observation period. The subsequent year control group stayed on the same PMSS levels over the similar period. The intervention was a significant predictor of PMSS reduction in a multiple regression analysis adjusted for age and sex, β = -1.93 (-3.47 to -0.38), P = 0.02. When we analysed the effects of self-development and discussion groups with the control group as reference, self-development group was the only significant predictor of PMSS reduction, β = -2.18 (-4.03 to -0.33), P = 0.02. There was no interaction with gender in our analysis. This implicates no significant difference between men and women concerning the effect of the self-development group. There was no reduction in general mental distress (SCL-5) over this period.

**Conclusion:**

A three-month follow-up showed that the intervention had a positive effect on perceived medical school stress among the students, and further analyses showed this was due to participation in self-development groups.

## Background

Stress and emotional disturbances among students at medical school are relatively common, and seemingly, this is a worldwide problem [1-4]. Studies indicate that specific stress factors related to medical school may induce mental health problems, and a decrease in life satisfaction among students [2,5-7]. Distress may affect their performance as students and later as carers for patients [8,9].

Interventions aimed at preventing mental health problems and prolonged negative stress during medical school have been recommended in several reports. Chew-Graham et al. recommend that support and mentoring by a tutor outside the students' working environment should be included in the preparation of physicians in order to identify and deal with professional stress in an appropriate manner [10]. Examples of interventions include: student counselling, support groups, and lectures focusing on stress reduction and coping strategies [11-14]. However, the intervention studies reported in the literature have limitations, such as small sample size, lack of control groups, and only addressing selected groups of students. Gender differences in mental health measures have been identified among medical students and doctors [3,15], and females constitute an increasing proportion of the total student population. Despite this, few studies have explored gender differences in students' response to interventions. Further, most studies have used qualitative self-reports, and few present quantitative data using validated instruments [16]. In addition, it has been suggested that voluntary recruitment may not reach all of those who need help [17]. To the best of our knowledge, no controlled studies have been published that have investigated the effect of mandatory group interventions for medical students.

Stress levels among medical students at different universities have been investigated using instruments focusing on the stressors within medical education [3,18]. The Perceived Medical School Stress (PMSS) scale is an example of such an instrument, which addresses and measures stress factors specifically related to medical school [19]. It consists of stressor items such as perceived threat, feelings of anonymity and isolation, and worries about schoolwork and competence. Lack of time for social activities and recreation, and worries about finances and accommodation are also included. In Norwegian medical students, a high level of perceived stress during medical school predicts undergraduate and postgraduate mental health problems that may require treatment [5,20]. Anxiety and depression in medical students have been assessed using a variety of instruments [6,21,22]. In Norway, various versions of the Symptom Check List (SCL-90) have been widely used to measure anxiety and depression [23] in the general population, in clinical groups and medical students [6,24,25]. A short version of this measure was used to capture general mental distress in our sample, and to identify if the intervention influenced such health problems.

With this background in mind, a group intervention aimed at preventing mental health problems among medical students was developed and carried out at the University of Bergen, Norway. The objective of the study was to compare a full class of students receiving specific interventions with another group that received no intervention. We investigated the course of perceived stress and general distress in the two groups over time to detect any possible short-term effects of the intervention.

## Methods

### Participants and procedures

Two subsequent classes of third-year medical students at the University of Bergen, Norway participated in this quasi-experimental study. Medical students in Bergen follow a "traditional" curriculum, with two years of pre-clinical studies, followed by four years of clinical training. Problem-based learning is not a part of the curriculum. The intervention took place in the initial months of the students' clinical training. The intervention group (n = 129) enrolled at the university in 2001, and a second group (n = 152) enrolled in 2002, acted as the control group. Both groups were assessed twice. The intervention group was assessed before (T1), and three months after end of intervention (T2), while the control group was assessed at the same points in time. The medical curriculum was identical for both groups.

The intervention was mandatory, but the participants could choose between two types of intervention. One option was self-development groups based on the model of therapy groups. The meetings focused on the students' positive resources and personal lives, and aimed at enhancing the participant's self esteem and personal insight. Another part of the programme involved identifying typical patterns of relationships that restricted their full capacity to relate to other people. The self-development group sessions were led by psychiatrists trained in group analytic treatment. Alternatively, the students could attend discussion groups where different topics relevant to medical studies and their later practice as doctors were discussed. Examples of issues discussed in these groups were: how to handle stress at work, balancing professional and private life, how to handle and communicate with difficult patients, and how to "break bad news" to patients and their relatives. Topics were selected by the students from a prepared list, or suggested by the students themselves. The discussion groups were lead by experienced general practitioners. The students were divided into groups of 8-10 participants. The format comprised 12 weekly group sessions, each lasting 90 minutes.

Participating in self-development or discussion groups was a mandatory part of the curriculum for medical students, similar to other mandatory courses at the Medical Faculty of the University of Bergen. However, to participate in the study concerning mental health was voluntary for all of the students, both in the intervention and control group. All students who chose to participate in the study signed a form of consent together with the first questionnaire. This form was sent together with an information letter describing the content, purpose and time dimension of the study. The letter also stated that the students would remain anonymous throughout and after the study. The same information was given orally in class for both groups. The questionnaires and information about the study were distributed by mail, with a postal reminder to non-responders.

Informed consent was obtained from 83.3% (234/281) of the students. Altogether, 75.6% (177/234) of those who consented to participate responded on all items on both occasions (T1 and T2). This constituted 63.0% (177/281) of the original sample. There were no statistically significant differences in gender or mean age between the self-development, discussion, and control groups.

The three groups' personality traits were also compared using the questionnaire Basic Character Inventory (BCI). BCI includes 27 items and capture Eysenck's Giant three personality trait dimensions: neuroticism, extraversion, and conscientiousness (low psychoticism) [7,26]. There were no statistically significant differences in distribution for the three traits between the self-development, discussion and control groups.

Ethical approval was obtained from the Regional Committee for Medical Research Ethics and the Norwegian Social Science Data Service, which are the authority of research ethics in Norway.

The programme was an integrated part of the curriculum and did not interfere with exams or holidays. Attendance was mandatory, but the students had the opportunity to be absent from two of the twelve group sessions, in case of sickness or other unplanned incidences. The leader of each group kept a list of attendance.

During the intervention period, group leaders held meetings to exchange experiences and discuss possible problems that may have occurred in the groups. For example, two students were advised to seek psychiatric treatment after such discussions among the leaders. The group leader meetings were administered and led by co-author K. I. S (Table 1).

**Table 1:** Age and gender of the medical students

	Self-development group	Discussion group	Control group	Total
**Age**	23.3 (2.3) (n = 54)	22.9 (2.1) (n = 50)	24.1 (4.2) (n = 111)	23.6 (3.4) (n = 215)

**Female**	63.6% (n = 35)	51.0% (n = 26)	61.1% (n = 69)	59.4% (n = 130)

**Male**	36.4% (n = 20)	49.0% (n = 25)	38.9% (n = 44)	40.6% (n = 89)

Gender and mean age (SD) before the intervention for medical students participating in the self-development group, discussion group, and the control group. The number of subjects vary for age (n = 215) and gender (n = 219) due to missing data. No differences were found between the groups according to the chi-squared test with a significance level set at 5%.

### Measures of distress

#### Perceived Medical School Stress

The level of perceived stress among students was measured using the Perceived Medical School Stress (PMSS) questionnaire [19], with minor adaptations to reflect the situation of Norwegian students [6], see Additional file 1. The PMSS questionnaire has shown a concurrent validity to symptoms of anxiety and depression among medical students [19], and a predictive validity on mental health problems in need of treatment four years after graduation from medical school [10].

Each of the 13 items has a five-point Likert scale, ranging from "strongly disagree = 0" to "strongly agree = 4". We used the total score of the items to indicate the level of stress among the students, where a high score indicated a high level of perceived stress. In the current study, the Cronbach's alpha coefficient for the entire group was 0.79 at T1. The 13 items of the PMSS scale were subjected to a principal component analysis with varimax rotation, including a scree plot evaluation. This confirmed three factors, in accordance with previously reported research [5]: "Medical school is cold and threatening", "Worries about work and competence", and "Worries about finance and accommodation".

#### General mental distress

The five-item edition (SCL-5) of the Hopkins Symptom Check List (SCL-90) was used to measure symptoms of mental distress among the students. The SCL-5 scale was developed and validated by Tambs and Moum to find anxiety and depressive symptoms [24,27], and it has previously been used among Norwegian medical students [28]. The questionnaire asks how much a person has been bothered by each of five specific symptoms over the past fourteen days. The symptoms listed are "Feeling fearful", "Nervousness or shakiness inside", "Feeling hopeless about the future", "Feeling blue" and "Worrying too much about things". Each item is rated on a five-point scale, from "not at all = 0" to "very much = 4". The mean item score indicated the level of mental distress. Cronbach's alpha coefficient for the entire group was 0.88 at T1.

#### Changes in scores over the observation period

To measure changes in perceived stress over the observation period, we computed a variable for the difference in PMSS scores between the two observation points: PMSS-Difference = the PMSS score at T2 minus the PMSS score at T1. A similar variable was calculated to show changes in SCL-5 distress from T1 to T2 (SCL-5-Difference).

#### Group intervention dummy variables

To study the effect of the two different interventions in the linear regression analysis, we computed two dummy variables to capture the effect of the three-category group variable. We used the control group as a reference, and named the dummy variables self-development group and discussion group respectively.

#### Statistics

The data were analysed using the SPSS statistical software package v15.0. The chi-squared test was applied to test for significant gender differences between the groups. One-way analysis of variance (ANOVA) was used to compare the mean age and mean sum of scores at T1 between the groups, and Tukey and Scheffé corrections were applied to the post-hoc tests. Student's paired t-test was applied to test for changes in PMSS mean sum score and SCL-5 mean item score from T1 to T2. Furthermore, the changes in PMSS and SCL-5 scores between T1 and T2 were tested using the mean of linear regression models. The significance level was set at 5% (95% confidence intervals) for all analyses.

## Results

**Figure 1:**
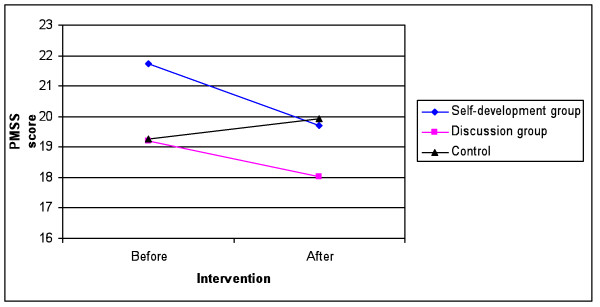
**Perceived Medical School Stress (PMSS) score before and after the intervention**. Total Perceived Medical School Stress (PMSS) score before and three months after the intervention for participants of the self-development and discussion group. Corresponding time periods for the control group. Change was significant for the self-development group in paired samples t-tests. P < 0.05.

The mean scores for PMSS and SCL-5 before (T1), and after (T2) the intervention and the change scores between T1 and T2 are shown in Table 2. No significant mean score differences were found between the groups at T1. The intervention group showed a significant reduction in mean total scores for PMSS (20.58 vs. 18.95, t = 2.61, P = 0.01). No such decline in PMSS was found for the control group over the similar period (19.26 vs. 19.94). When the two intervention groups were analysed separately, only the self-development group showed a significant decrease in mean total scores for PMSS (21.72 vs. 19.72, t = 2.30, P = 0.03). There was no such decline in SCL-5 over the observation period. Figure 1 shows the PMSS scores before, and three months after the intervention for the three groups. We studied age, gender, intervention and the group intervention dummies as predictors of PMSS-Difference in the sample (Table 3). Unadjusted predictors of PMSS-Difference in a bivariate regression were: intervention, β = -2.31 (-3.85 to -0.76), P < 0.01, age, β = 0.27 (0.03 to 0.51), P = 0.03, and self-development group, β = -2.13 (-3.90 to -0.36), P = 0.02. In the first adjusted multiple regression analysis we tested the effect of the intervention versus the control group when adjusted for age and sex. The intervention was a significant predictor, β = -1.93 (-3.47 to -0.38), P = 0.02. In the second multiple regression analysis, we included group intervention dummies in order to test for any differences between the intervention groups. Self-development group was the only significant predictor with the control group as the reference, β = -2.18 (-4.03 to -0.33), P = 0.02. There was a trend in our results showing male students in the self-development groups having a greater decrease in PMSS score than their male peers in the discussion and control groups did, but there was no interaction between gender and the self-development group. This means that there were no significant gender differences concerning the effect of the self-development group.

**Table 2:** Changes in Perceived Medical School Stress (PMSS) and Symptom Check list (SCL-5) score over the period

	Self-development group (n = 47)		Discussion group (n = 39)		Control group (PMSS n = 93, SCL-5 n = 94)	
	
	T1	T2	T1	T2	T1	T2
**PMSS**	21.72 (19.46, 23.99)	19.72* (17.43, 22.02)	19.21 (17,12, 21.29)	18.03 (15.93, 20.12)	19.25 (17.90, 20.61)	19.94 (18.48, 21.39)

**Change in PMSS score**	-2.00 (-1.18, -0.25)		-1.18 (-3.00, 0.65)		-0.68 (-0.28, 1.63)	

**SCL-5**	0.79 (0.62, 0.96)	0.81 (0.61, 1.02)	0.76 (0.50, 1.03)	0.62 (0.40, 0.83)	0.74 (0.58, 0.89)	0.77 (0.64, 0.92)

**Change in SCL-5 score**	0.03 (-0.19, 0.24)		-0.15 (-0.36, 0.06)		0.04 (-0.07, 0.16)	

* Changes were significant in paired samples t-tests. P < 0.05. Mean sum scores and mean change scores for Perceived Medical School Stress (PMSS) n = 179, and mean item score and mean change scores for Symptom Check list (SCL-5), n = 180, for students responding at both T1 and T2. The 95% confidence interval for the mean and difference is given in parentheses.

**Table 3:** Predictors for changes in PMSS scores among students at T2. N = 177 in the multivariate analysis

	Bivariate analyses			Multivariate analysis		
	
	Crude β	95% CI	P value	Adjusted β	95% CI	P value
**Age **(n = 177)	0.27	0.03, 0.51	0.03	0.22	-0.02, 0.47	0.07

**Gender (male = 1)** (n = 179)	0.13	-1.48, 1.73	0.88	0.25	-1.33, 1.82	0.76

**Self-development group **(n = 179)	-2.13	-3.90, -0.36	0.02	-2.18	-4.03, -0.33	0.02

**Discussion group** (n = 179)	-0.96	-2.87, 0.95	0.32	-1.62	-3.59, 0.35	0.11

## Discussion

The most important finding of this study was that the intervention was a significant and independent predictor of the decrease in PMSS over the observation period. When we tested the effects of each of the intervention groups, only the self-development group was a significant predictor of the reduction in PMSS. However, we found no effect of the intervention on general mental distress, as measured by the SCL-5 score. This suggests that the intervention affected the specific stress related to medical school, rather than general mental distress. The intervention, and in particular the self-development programme, reduced the specific factors related to attending medical school and negative attitudes towards medical training. Examples of such stressors that the PMSS seems to capture include: lack of thriving in the study situation, a sense of opposition towards teachers and the curriculum, a feeling of being controlled too much, of having too little space for personal interests, and not being seen as an individual.

Two recent papers have studied the effects of self-selected interventions among medical students. Both Finkelstein et al[12] and Rosensweig et al[13] report beneficial effects on mental health from a voluntary stress reduction class. The participating students had higher initial scores on mental health parameters than their peers who did not seek help and no gender differences were reported [12,13]. We believe that our mandatory intervention design may be important. In planning the study, we assumed that some students would not participate voluntarily in a group intervention that had elements of psychotherapy, and that this would particularly apply to male students. This assumption was however not supported in our data. Nevertheless, the importance of a student's opportunity to choose between the two different types of group should not be underestimated.

In a qualitative evaluation carried out among the students at the last meeting of the group sessions, the discussion groups were generally evaluated as being more popular. From this, we may have expected this type of group to be more effective than the self-development groups were [29]. However, in contrast, the self-development groups seem to be more effective. Why might this be?

The self-development groups may have given the students an experience of being seen as individuals, and that their personal recourses were acknowledged as valuable in becoming a good physician. Some of the students said that they experienced an openness about personal problems that was a new experience at medical school. They also mentioned that building a network among their peers was valuable, as the group members became closer to each other and could share problems. Thus, they had a feeling of a safety net that would help them solve new problems. They also thought it would be easier for them to talk to colleagues about personal or professional problems, and this lowered the threshold for them to seek professional help in the future. They simply learned that it was acceptable to have problems, since their peers had disclosed problems of their own. The participation in the groups may have helped the students to tolerate insecurity and ambiguity, a common aspect of all medical practice. This is consistent with reports from previous self-development groups of volunteer female medical students at the University of Bergen [30].

Similar comments were also made by students of the discussion groups. The leaders of the discussion groups reported a high level of personal involvement from the students, and that the group discussion often dealt with private and personal issues [29]. This in spite of the more rigid and preset structure of the meetings.

An important and specific part of the self-development groups was learning about relational patterns from their past, which hindered a more flexible attitude towards peers, teachers, and patients. This may contribute to a more robust and sustainable effect of the self-development groups than that of the more "external reality based" discussion groups. But this remains to be investigated by longer term follow- up of our cohort.

Since the students themselves chose which particular group intervention to join, even though participating in a group was mandatory, it seems that this self-selection was especially successful for those who chose the self-development groups. These students may have had high levels of insight into the type of help they needed. Another factor of great importance for the positive outcome is the specific qualifications of the group leaders of the self-development group. This factor may restrict the practical applicability of the programme in other medical schools, since such highly qualified psychiatrists with group-analytical training are not easy to recruit.

High levels of PMSS have been found to predict mental health problems that require treatment, and hence, the PMSS score may represent a vulnerability measure [5,20]. Stress may affect academic performance negatively [8], and increase the chances of developing depression [6,19]. However, only long-term follow-ups will show whether these initial results are stable throughout and following graduate school, implying improved mental health, and improved management of the specific stressors involved in medical practice.

The evaluation after the groups had ended showed that most students appreciated the groups, even though they were mandatory [29]. On the other hand, some students said that they lacked interest in the groups, and that they were not motivated to participate because of the mandatory requirement. The students showed ambivalent attitudes towards making the group programme a mandatory part of the curriculum on a permanent basis. Although neither of the two interventions was defined as treatment, students who participated in the groups said that one positive aspect of making them mandatory would be that teachers could pick out students who needed to seek professional help. The self-development and discussion groups have previously been voluntary for medical students in Bergen. At that time, however, only half of the students participated in the voluntary groups, and these were mostly female students [29].

The intervention and design applied in this study has several strengths and limitations. The prospective design with pre-post intervention measures and a control group are major strengths. The mandatory intervention and that the group leaders kept track of any student absent ensured a good compliance, and that we reached all students in need of help. To our knowledge, few studies have evaluated this type of mandatory intervention programme using a control group. The instruments applied have also been validated for the Norwegian population. One important limitation of our study is the lack of a randomized, controlled trial design. The intervention groups and the control group were from two different student classes and were assessed during two different calendar years. It is possible that the pressures on the intervention and control groups have been different, so also the motivation to participate in the study. This design implies a risk of a confounding cohort effect. There were however no known changes in the curriculum from the one year to the other. Further, there were no significant differences in general mental distress and medical school stress at baseline between the two cohorts. Though, it is a weakness of our study that it was not conducted according to the design of a randomized controlled trial, and as a consequence we should be cautious to make too firm conclusions. The positive effects of such group interventions should be further explored with a randomized, controlled trial design. In our study a randomization would not have given the students the opportunity to choose between the two different types of groups. This would have forced unmotivated students to participate in self-development groups, and perhaps hindered the group process. On the other hand, even without randomization, any mandatory intervention runs the risk that unmotivated students may be included. Responses from both leaders and students confirmed that this was the case in some groups. This might have reduced the positive effects of the intervention of the present study. It may also be a limitation that the study was based on self-report measures. This may lead to less reliable reporting of mental distress, for example, an underreporting of such distress.

## Conclusions

This is one of the first controlled group intervention studies to show an effect on stress among a whole class of medical students. The self-selected self-development intervention may beneficially affect students' perception of specific stressors related to medical school. The effects of such group interventions should however be studied in other samples of medical undergraduates, and preferably with a randomized, controlled trial design.

## Competing interests

The authors declare that they have no competing interests.

## Authors' contributions

MH drafted the manuscript and performed the statistical analyses. RT contributed to the interpretation and the analyses of the data, and revised the manuscript. KIS participated in the conception and design of the study and revised the manuscript. BH participated in the conception and design of the study and helped to draft and revised the manuscript. All authors approved the final manuscript.

## Pre-publication history

The pre-publication history for this paper can be accessed here:

http://www.biomedcentral.com/1472-6920/10/23/prepub

## Supplementary Material

Additional file 1 Perceived Medical School Stress Questionnaire Click here for file
