# Supplementary material for: Self-development groups reduce medical school stress: a controlled intervention study
Source: BMC Med Educ. 2010 Mar 16;10:23. doi: 10.1186/1472-6920-10-23 (PMC2847570; doi:10.1186/1472-6920-10-23)
Supplement: Additional file 1 — Perceived Medical School Stress Questionnaire [file 1472-6920-10-23-S1.DOC]

1. Medical school fosters a sense of anonymity and feelings of isolation among the students
2. I am concerned that I will not be able to endure the long hours and responsibilities associated with clinical training and practice
3. I do not know what the faculty/administration expect of me
4. Medical training controls my life and leaves too little time for other activities
5. I am concerned that I will unable to master the entire pool of medical knowledge
6. This medical school is fostering a physician role at the expense of one’s personality and interests
7. Medical school is more competitive than I expected
8. The attitude of too many of the faculty is that students should be subjected to ‘baptism of fire’
9. The majority of students feel that success in medical school is in spite of the administration rather than because of it
10. Medical school is cold, impersonal and needlessly bureaucratic
11. Medical school is more of a threat than a challenge
12. Personal finances are a source of concern to me
13. Accommodation is a source of concern to me
